# Hyperuricemia and the risk for coronary heart disease morbidity and mortality a systematic review and dose-response meta-analysis

**DOI:** 10.1038/srep19520

**Published:** 2016-01-27

**Authors:** Min Li, Xiaolan Hu, Yingli Fan, Kun Li, Xiaowei Zhang, Wenshang Hou, Zhenyu Tang

**Affiliations:** 1 Department of Neurology, The Second Affiliated Hospital to Nanchang University, Nanchang 330006, People’s Republic of China; 2 Department of Anaesthesiology, The Second Affiliated Hospital to Nanchang University, Nanchang 330006, People’s Republic of China; 3 Department of Cardiology, The Second Affiliated Hospital to Nanchang University, Nanchang 330006, People’s Republic of China

## Abstract

Considerable controversy exists regarding the association between hyperuricemia and coronary heart disease (CHD). Therefore, we performed a systematic review and dose-response meta-analysis of prospective studies to examine the controversy. Prospective cohort studies with relative risks (RRs) and 95% confidence intervals (CIs) for CHD according to serum uric acid levels in adults were eligible. A random-effects model was used to compute the pooled risk estimate. The search yielded 29 prospective cohort studies (n = 958410 participants). Hyperuricemia was associated with increased risk of CHD morbidity (adjusted RR 1.13; 95% CI 1.05 to 1.21) and mortality (adjusted RR 1.27; 95% CI 1.16 to 1.39). For each increase of 1 mg/dl in uric acid level, the pooled multivariate RR of CHD mortality was 1.13 (95% CI 1.06 to 1.20). Dose-response analysis indicated that the combined RR of CHD mortality for an increase of 1 mg uric acid level per dl was 1.02 (95% CI 0.84 to 1.24) without heterogeneity among males (*P* = 0.879, *I*^*2*^ = 0%) and 2.44 (95% CI 1.69 to 3.54) without heterogeneity among females (*P* = 0.526, *I*^*2*^ = 0%). The increased risk of CHD associated with hyperuricemia was consistent across most subgroups. Hyperuricemia may increase the risk of CHD events, particularly CHD mortality in females.

Cardiovascular disease (CVD) is one of the most common noncommunicable diseases which is forecasted to be the major cause of morbidity and mortality in most developing nations by 2020^1^. In 2011, 375295 Americans died of coronary heart disease (CHD). Each year, an estimated ≈635000 Americans have a new coronary attack and ≈300000 have a recurrent attack. Approximately every 34 seconds, 1 American has a coronary event, and approximately every 1 minute 24 seconds, an American will die of one^2^. The prevention of CVD (especially CHD) is thus clearly a major public health issue. In recent decades, concern has mounted regarding the premature mortality and morbidity associated with CHD, with growing interest in altering risk factors and reversing this global epidemic. Among the novel risk factors for CHD, nutritional factors have aroused particular attention. Prospective observational studies have been used to quantify the total effects of dietary habits on CHD^3^,^4^,^5^. Although the effect of individual components or interactions between dietary habits is still largely unknown or even misconstrue actual total impact on vascular health, elevated serum uric acid levels may explain some of this harmful effect^6^.

Serum uric acid is a final enzymatic product of purine metabolism^7^,^8^. Although hyperuricemia is no universally accepted definition, it is generally defined as serum urate concentration in excess of 6.8 mg/dl^9^. In the Atherosclerosis Risk in Communities (ARC) Study^6^, after 3 and 9 years of follow-up, the odds ratio for developing CHD was 2.59 for participants who had a serum acid level >9.0 mg/dl. Moreover, several meta-analyses of observational studies have found that hyperuricemia could significantly increase the risk of CHD events^10^,^11^,^12^. These studies were restricted by heterogeneous with respect to sample size. Furthermore, previous meta-analyses did not assess important differences in the exposure type, such as relevant subgroups (for example, sample size, and other characteristics that may contribute to inconsistencies in the literature); and scientific rigour (for example, the quality of the study and duration of follow-up). Additionally, recent studies involving relationship between hyperuricemia and risk of CHD have been published from then on^13^,^14^,^15^,^16^, results from cohort studies are still controversial. Furthermore, whether any dose-response relation exists between hyperuricemia and risk of CHD is unknown. Therefore, we systematically reviewed and meta-analysed available studies to quantify the associations between hyperuricemia and risk of CHD morbidity and mortality based on identified prospective cohort studies. We also conducted a dose-response analysis for the trend estimation.

## Methods

### Data Sources and Searches

We performed a systematic search of PubMed (MEDLINE) and EMBASE through August 23, 2015. The following key words were used in our search strategies: (“hyperuricemia” OR “uric acid” OR “urate”) AND (“coronary heart disease” OR “cardiovascular disease” OR “ischemic heart disease” OR “myocardial infarction” OR “coronary artery disease” OR “coronary disease” OR “angina pectoris” OR “unstable angina”) AND (“follow-up studies” OR “prospective studies” OR “cohort studies” OR “longitudinal studies” OR “epidemiological studies” OR “observational studies”). We restricted the search to human studies. No restrictions were imposed on language of publications. In addition, we scrutinized possible eligible references from relevant original papers and review articles to identify potential publications. The search strategy was conducted according to the recommendations of the Meta-analysis of Observational Studies in Epidemiology (MOOSE) (Appendix 1)^17^.

### Study Selection

Studies were selected for the meta-analysis if they fulfilled the following entry criteria: (1) original studies (eg, not review articles, meeting abstracts, editorials, or commentaries); (2) prospective cohort design (eg, not cross sectional design, case-control design); (3) the exposure of interest was hyperuricemia or elevated serum uric acid level; (4) the outcomes were CHD morbidity and (or) mortality; (5) reported adjusted risk estimates for the association between hyperuricemia assessed as serum, and CHD morbidity and/or mortality; (6) longer than one year of follow-up; and (7) participants were free of kidney disease or CHD at study entry. Additionally, we excluded animal studies, clinical trials, commentaries and letters without sufficient data. If data were reported more than once, we included the study with the longest follow-up time. For studies that expressed data separately for males and females, we considered the analysis for each sex as an independent comparison and extracted data separately.

### Data Extraction and Quality Assessment

Data were carried out independently by two authors (XZ, XH, and WH) using a standard electronic sheets and cross-check to reach a consensus. For each study, the following information was abstracted: name of the first author, year of publication, country where the cohort was performed, geographical location, size of the cohort and proportion of males, age range or mean, duration of follow-up, methods used to assess hyperuricemia and ascertain CHD, number of cases, and adjusted covariates. All data were extracted from the published papers. If necessary, the primary authors were contacted to retrieve further information.

The Newcastle-Ottawa Scale (NOS) was used to assess the quality of studies^18^. The quality of cohort studies were evaluated in the following three major components: selection of the study group (0–4 stars), quality of the adjustment for confounding (0–2 stars) and assessment of outcome in the cohorts (0–3 stars). A higher score represents better methodological quality. The full score was 9 stars. Studies were graded as the high-quality if they met >8 awarded stars.

### Data Synthesis and Statistical Analysis

Within each study, we used multivariate-adjusted outcome data (expressed as relative risks [RRs] and 95% confidence intervals [CIs]) for risk estimates. The hazard ratios (HRs) were considered equivalent to RRs, we converted these values in every study by taking their natural logarithms and calculating standard errors and corresponding 95% CIs. RRs and their standard errors were pooled with the DerSimonian and Laird random effects model, which takes into account both within-study and between-study variabilities^19^. When some studies included in our meta-analysis used the International System (IS) of units to report levels of serum uric acid, we converted those to the conventional units, using a conversion rate of 16.81 (1 mg/dL = 59.48 μmol/L). If the result on CHD was not available, we used data from ischemic heart disease, myocardial infarction or angina pectoris (in the sequential order) as a surrogate for CHD.

For the dose-response analysis, the generalized least square for trend estimation method described by Greenland and Longnecker^20^ and Orsini *et al.*^21^,^22^ was used to calculate study-specific slopes (linear trends) and 95% CIs. The method requires the distributions of cases and person years for exposure categories, and median/mean of serum uric acid levels for each comparison group. We assigned the midpoint of the upper and lower boundaries of each comparison group to determine mean uric acid levels if the median or mean intake was not provided. If the lower or upper boundary for the lowest and highest category, respectively, was not reported, we assumed that the boundary had the same amplitude as the closest category. Additionally, we first created restricted cubic splines with 3 knots at percentiles 25%, 50%, and 75% of the distribution^23^. A *P* value for nonlinearity was calculated by testing the null hypothesis that the coefficient of the fractional polynomials component is equal to zero.

Heterogeneity among studies was evaluated using the chi-square test based on Cochran’s Q test and *I*^*2*^ statistic at *P* < 0.10 level of significance^19^, and quantification of heterogeneity was made by the *I*^*2*^ metric, which describes the percentage of total variation in point estimates that is due to heterogeneity rather than chance^24^. We considered low, moderate, and high degrees of heterogeneity to be *I*^*2*^ values of 25%, 50%, and 75%, respectively^24^,^25^. To explore possible explanations for heterogeneity and to test the robustness of the association, we conducted subgroup analyses based on the quality of the study (high quality (9) v lower quality (<9), length of follow-up (>10 years v ≤ 10 years), sex (male and female included v female only v male only), number of participants (>5000 v ≤ 5000), and Geographical area (United States versus Asian versus European). Meta-regression and sensitivity analyses were also conducted. We performed the Begg rank correlation test and Egger’s regression test to visualize a possible asymmetry^26^,^27^,^28^. Funnel plots were also used to assess the publication bias. We used a trim and fill algorithm if possible publication bias was detected to identify and correct for the asymmetry of funnel plot from publication bias and provide an adjusted summary RR based on all the studies, including the estimated missing studies^29^. On the other hand, when the limited number (below 10) of studies was included in each analysis, publication bias was not assessed^30^.

All the statistical analyses were performed in Stata 12 (Stata Corp, College Station, TX). A threshold of *P* < 0.1 was used to decide whether heterogeneity or publication bias was present^27^. In other ways, *P* values were 2-sided and *P* < 0.05 was considered statistically significant.

## Results

### Literature Search

**Figure 1:**
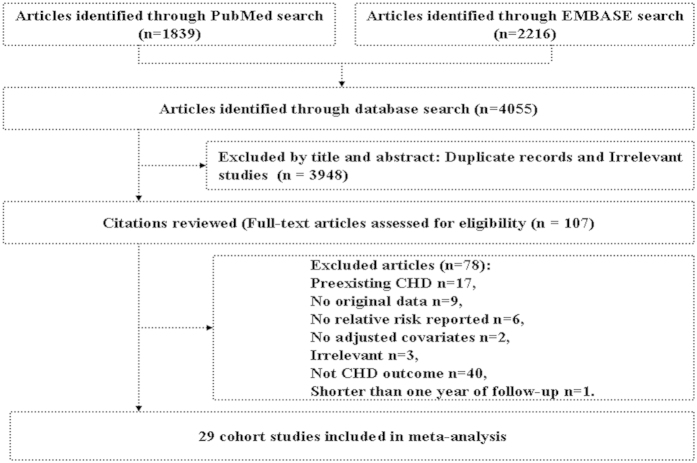
Process of literature search and study selection. CHD: coronary heart disease.

In total the search strategy retrieved 4055 unique articles (1839 articles from PubMed and 2216 articles from EMBASE) (Fig. 1). After exclusion of duplicate records and studies that did not fulfill our inclusion criteria, 107 articles remained, and we further evaluated the full texts of these 107 publications. Of these, we excluded 78 studies as follows. Six articles were excluded owing to lack of sufficient data for estimation of RRs. Nine articles were excluded because no original data could be extracted (comment, review, or cross sectional studies). Forty articles were excluded owing to not an outcome of interest. Seventeen articles were excluded because of preexisting CHD at study entry. Three articles were excluded because we deemed irrelevant. Two articles were excluded owing to no adjusted covariates. Another one was excluded because duration of follow-up was shorter than one year. In aggregate, a total of 29 prospective cohort studies representing data from 958410 participants were included in this meta-analysis^10^,^13^,^14^,^15^,^16^,^31^,^32^,^33^,^34^,^35^,^36^,^37^,^38^,^39^,^40^,^41^,^42^,^43^,^44^,^45^,^46^,^47^,^48^,^49^,^50^,^51^,^52^,^53^,^54^. In addition, ten studies that expressed data separately for males and females^10^,^15^,^34^,^35^,^37^,^38^,^43^,^45^,^47^,^53^. According to the study selection criteria, we considered the analysis for each sex as an independent comparison and extracted data separately. Thus, our meta-analysis included 36 comparisons.

### Study Characteristics

Table 1 show the characteristics and main outcomes extracted from the included studies, all 29 studies (14 for CHD morbidity^10^,^13^,^14^,^31^,^32^,^33^,^34^,^35^,^36^,^37^,^38^,^39^,^40^,^41^ and 15 for CHD mortality^15^,^16^,^42^,^43^,^44^,^45^,^46^,^47^,^48^,^49^,^50^,^51^,^52^,^53^,^54^) were prospective cohort designs and participants who were free of CHD at baseline^10^,^13^,^14^,^15^,^16^,^31^,^32^,^33^,^34^,^35^,^36^,^37^,^38^,^39^,^40^,^41^,^42^,^43^,^44^,^45^,^46^,^47^,^48^,^49^,^50^,^51^,^52^,^53^,^54^. In total, the included studies consisted of 958410 participants. Of these participants, we identified 31643 of CHD occurred during follow-up periods ranging from 5.4 to 24.9 years (median of 11.7 years)^44^,^47^. Among 29 articles, nine cohorts were conducted primarily in the United States^16^,^31^,^32^,^34^,^35^,^39^,^42^,^43^,^50^, eight articles were done in Asian countries (China, Korea, Israel, Japan)^15^,^37^,^40^,^44^,^46^,^47^,^49^,^54^ and twelve cohorts were from European countries (Germany, Italy, Iceland, the Netherlands, Norway, Belgium, Greece, Sweden, Austria)^10^,^13^,^14^,^33^,^36^,^38^,^41^,^45^,^48^,^51^,^52^,^53^. All except one were written in English^45^. The number of participants ranged from 960 in the Monitoring Trends and Determinants in Cardiovascular Diseases Augsburg (MONICA) cohort by Liese *et al.*^33^ to 417734 in the Apolipoprotein MOrtality RISK study (AMORIS) by Holme *et al.*^53^. Ten studies included both male and female^10^,^15^,^34^,^35^,^37^,^38^,^43^,^45^,^47^,^53^, nine studies included only male^32^,^33^,^39^,^41^,^44^,^46^,^49^,^50^,^51^, two cohorts included only female^42^,^52^. The age of participants ranged from 20 to 85 years^15^,^53^. The definition of hyperuricemia ranged from 5.6 to 7.7 mg/dl in males^37^,^49^ and from 4.7 to 7.0 mg/dl in females^10^,^54^. In most of the studies, CHD events were assessed by medical records and/or death certificates based on International Classification of Diseases (ICD) codes-8,9,10. All except two studies provided multivariate-adjusted risk estimates (e.g., age, sex, body mass index, smoking, cholesterol, *et al.*), the majority (93%) of included studies were of high quality (9 stars) (Supplemental table A in appendix 2)^32^,^44^.

### Hyperuricemia and Risk of CHD Morbidity

**Figure 2:**
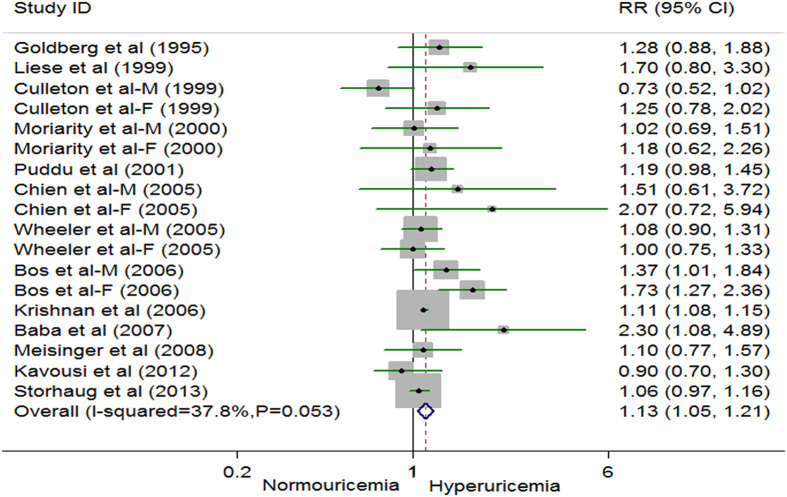
Random effects analysis of multivariate risks of coronary heart disease morbidity associated with hyperuricemia. F: female, M: male, RR: relative risk. CI: confidence interval.

18 comparisons from thirteen studies reported an association between hyperuricemia and risk of CHD morbidity, with 6666 CHD cases and 70382 participants^10^,^13^,^14^,^32^,^33^,^34^,^35^,^36^,^37^,^38^,^39^,^40^,^41^. Overall, the random effects model suggested a positive association; the pooled RR was 1.13 (95% CI 1.05 to 1.21) (Fig. 2). There was moderate study heterogeneity (*P* = 0.053, *I*^*2*^ = 37.8%). Additionally, slight publication bias was observed from the Begg (*P* = 0.096), Egger regression tests (*P* = 0.267), and the funnel plot (see supplemental Figure A in appendix 3). Trim and fill analysis, however, did not change the result (see supplemental Figure B in appendix 3). When a single study involved in the meta-analysis was deleted each time, the results of meta-analysis remained non-significant. A multivariate meta-regression analysis was performed to further investigate the effect of three study-level characteristics (geographical area, sex, length of follow-up and the quality of the study) on the risk of CHD morbidity. None of the regression coefficients was statistically significant (see supplemental table B in appendix 2). Among 18 comparisons, five comparisons (3 for males and 2 for females) were eligible for the dose-response analysis of hyperuricemia and risk of CHD morbidity^34^,^35^,^41^. Using a restricted cubic splines model, we found no evidence of a curve linear association (*P* = 0.561; *P* = 0.299 for non-linearity respectively, see supplemental Figures C and D in appendix 3). Dose-response analysis found no associations with risk of CHD morbidity per 1 mg/dl increment of serum uric acid level RR 0.93, 95% CI 0.72 to 1.20, *I*^*2*^ = 34.3%) among males^34^,^35^,^41^ and (RR 1.22, 95% CI 0.83 to 1.80, *I*^*2*^ = 0%) among females (see supplemental Figure E in appendix 3)^34^,^35^.

### Hyperuricemia and Risk of CHD Mortality

**Figure 3:**
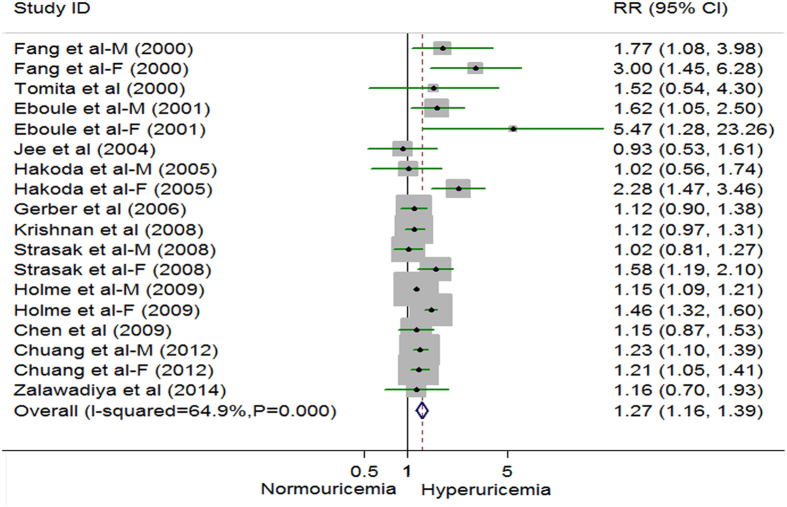
Random effects analysis of multivariate risks of coronary heart disease mortality associated with hyperuricemia

Thirteen studies exported an association between hyperuricemia and risk of CHD mortality, with 24198 CHD cases and 876584 participants^15^,^16^,^43^,^44^,^45^,^46^,^47^,^49^,^50^,^51^,^52^,^53^,^54^. Using a random effects model summarizing all 18 comparisons, participants with hyperuricemia, compared with normouricemia, experienced a significant increased risk for death of CHD (RR 1.27, 95% CI 1.16 to 1.39) (Fig. 3), although significant heterogeneity was detected (*P* = 0.000, *I*^*2*^ = 64.9%). The funnel plot was symmetry, no evidence of substantial publication bias was observed from the Begg (*P* = 0.225) and Egger regression tests (*P* = 0.102) (see supplemental Figures F and G in appendix 3). When a single study involved in the meta-analysis was also deleted each time, the results of meta-analysis remained non-significant. Moreover, a multivariate meta-regression analysis was also performed to further investigate the effect of four study-level characteristics (geographical area, sex, length of follow-up and the quality of the study) on the risk of CHD mortality. None of the regression coefficients was statistically significant (see supplemental table B in appendix 2). Among 18 comparisons, six comparisons (4 for males and 2 for females) were eligible for the dose-response analysis of hyperuricemia and risk of CHD mortality^43^,^44^,^46^,^47^,^51^. Using a restricted cubic splines model, we found no evidence of a curve linear association (*P* = 0.819 for non-linearity and *P* for linear trend <0.001 respectively, see supplemental Figures H and I in appendix 3). Dose-response analysis found no associations with risk of CHD mortality per 1 mg/dl increment of serum uric acid level (RR 1.02, 95% CI 0.84 to 1.24, *I*^*2*^ = 0%) among males (see supplemental Figure J in appendix 3)^44^,^46^,^47^,^51^. However, dose-response analysis found significant association with risk of CHD mortality per 1 mg/dl increment of serum uric acid level (RR 2.44, 95% CI 1.69 to 3.54, *I*^*2*^ = 0%) among females (see supplemental Figure J in appendix 3)^43^,^47^.

**Figure 4:**
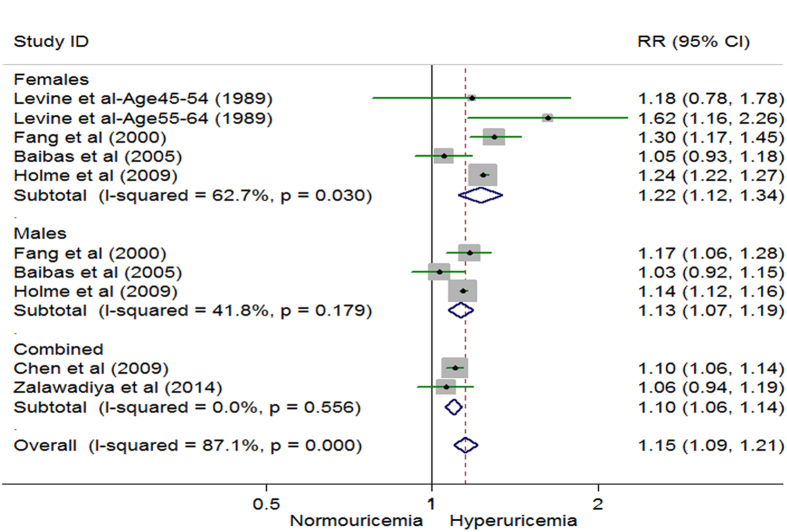
Random effects analysis of multivariate risks of coronary heart disease mortality associated with an increase of 1 mg/dl in serum uric acid level. Combined: studies which did not have sex specific data

We paid close attention to each increase of 1 mg/dl in uric acid level. The overall pooled multivariate RR for CHD mortality was 1.15 (N = 5 studies, 95% CI 1.09 to 1.21) (Fig. 4)^16^,^42^,^43^,^48^,^54^. The gender specific RRs for each increase of 1 mg/dl in serum uric acid level were similar, but a more pronounced increased risk for CHD mortality in females was observed (Fig. 4). We did not assess publication bias owing to the limited number of included studies.

### Subgroup Analyses

To examine the stability of the primary results, we carried out subgroup analyses. The association between hyperuricemia and risk of CHD morbidity and mortality was similar across most subgroups, which were separately defined study quality, length of follow-up, sex, geographical area, and number of participants affected the results. The summary estimates of relative risks from each category were pooled (see supplemental table C in appendix 2).

## Discussion

The results of this meta-analysis demonstrate that hyperuricemia is prospectively associated with a significantly increased risk of CHD morbidity and mortality. Furthermore, the association persisted and remained statistically significant across most subgroups stratified by various study and participant characteristics. The overall risk of CHD mortality increased 15% for each increase of 1 mg/dl of uric acid, this association seems to be stronger for females that males. Dose-response analyses indicated higher risk of CHD mortality per 1 mg/dl increment of serum uric acid level in females, but no significant trend for males and CHD morbidity. These dose-response results should be interpreted with caution, because the limited number of studies was included in each analysis.

Over the past decades, extensive prospective studies have reported the association of hyperuricemia combined with CVD risk (including CHD and stroke)^55^,^56^,^57^,^58^,^59^. However, the role of serum uric acid level in CHD is still controversial. Some of the studies failed to find the association between hyperuricemia and risk of CHD^10^,^13^,^14^,^16^,^32^,^33^,^34^,^35^,^36^,^37^. However, Chuang and colleagues analysed data from a large Chinese cohort with 7.33 years of follow-up and found that hyperuricemia was independently associated with the development of ischemia heart disease^15^. Similar to previous analysis in the Apolipoprotein Mortality RISk study (AMORIS), the result from 417734 participants also supported an positive association between hyperuricemia and risk of CHD both in males and in females^53^. But these studies did not assess or adjust for exercise, education level and other important risk factors such as use of antihypertensive medication^48^. Diuretics are frequently used to control hypertension, and it had been reported to elevate the levels of serum uric acid.

The results from the Framingham Heart Study indicated that uric acid did not have a causal role in the development of CHD, independent of use of diuretics^34^. However, in the First National Health and Nutrition Examination Survey (NHANES I) Epidemiologic Follow-up Study found that the serum uric acid level was predictive of mortality from ischemic heart disease among females, but no associations were seen among males. These associations were independent of use of antihypertensive agents and diuretics^31^. Reasons for these conflicting finding were uncertain, but the large intraindividual variation in levels of uric acid, which approaches the variability among persons, may have weakened the strength of the associations observed in several studies^60^.

Other than hypertension and use of antihypertensive medication or diuretics, age is an important role of other factors such as smoking, alcohol consumption and body mass index. In the Chicago Heart Association Detection Project, CHD deaths were associated with serum uric acid levels in the 55–64 year old group, but not in the 45–54 year group^42^. But, in the Apolipoprotein MOrtality RISk study (AMORIS), the results supported positive associations between hyperuricemia and risk of CHD. These associations were independent of age^53^. On the other hand, Freedman *et al.*^31^ found that the serum uric acid level was predictive of mortality from ischemic heart disease among females with an age ≥55 years, but no associations were seen among males and patients with an age <55 years.

Several plausible biological mechanisms have been proposed to explain abovementioned association. Uric acid may stimulate vascular smooth cell proliferation, reduce vascular nitric oxide production, diminish vascular nitric oxide activity, and link to insulin resistance^61^,^62^. It has been demonstrated that uric acid independently associated with the levels of C-reactive protein, interleukin-6, interleukin-18, and tumour necrosis factor-alpha^63^. On the other hand, inflammation has been considered a key factor during the development of coronary artery disease, via atherogenesis and thrombogenesis^64^. Moreover, serum uric acid has been positively associated with arterial intima-media thickness, a precursor to atherosclerosis and thus to CHD^65^. In aggregate, results in metabolic or endocrine changes. Through sympathetic activation and elevated levels of inflammatory cytokines, may link the development of hyperuricemia with the pathogenesis of CHD events.

Heterogeneity between studies was found, which did not alter much in the subgroup analyses. There are differences in serum uric acid levels between males and females among different countries. Therefore, within the subgroup analysis we examined sex and geographical area as possible sources of heterogeneity. Females were more likely than males to have higher prevalence of hypertension and high LDL cholesterol, and menopause among females made higher risk than males among in Asian^66^,^67^. We also examined study quality, number of participants, and length of follow-up as possible sources of heterogeneity, these did not appreciably alter much heterogeneity between studies. Of course, the observed heterogeneity could be attributable to differences in environmental factors, methodological factors in design, and how the studies were conducted. Sensitivity analyses showed that exclusion of these only age-adjusted studies did not obviously alter the pooled relative risks^32^,^44^. Thus, the presence of heterogeneity calls for caution in interpreting the current meta-analysis findings. However, although the meta-regression could not explain the level of heterogeneity, in interpreting the results, several differences between the studies are worth discussing.

To date, it is generally defined as serum urate concentration exceeding the limit of solubility (about 6.8 mg per deciliter)^9^. Moreover, it is a common biochemical abnormality that reflects supersaturation of the extracellular fluid with urate and predisposes affected persons to gout. As mentioned earlier, the definition of hyperuricemia ranged from 5.6 to 7.7 mg/dl in males and from 4.7 to 7.0 mg/dl in females. Thus, these ambiguous measurements may underestimate or overestimate true associations between hyperuricemia and risk of coronary artery disease events. Another possible explanation for the differences between the studies might be the classification of outcomes. Coronary artery disease’s definition was inconsistent: the end point from six studies was myocardial infarction^14^,^33^,^38^,^39^,^41^,^53^; four were ischemia heart disease^15^,^31^,^43^,^46^. If they were included with an uniform definition, the evidences might be stronger.

Compared with the previous meta-analyses^10^,^11^,^12^, our study has several strengths. Our meta-analysis included prospective cohort studies with long duration of follow-up and large sample size, which significantly increased the statistical power to detect potential associations and avoided the influence of recall and selection bias. In addition, to examine the shape of these possible associations, we investigated a dose-response relation hyperuricemia and risk of CHD morbidity and mortality. Furthermore, we used models adjusting for most established risk factors and conducted subgroup analyses and sensitivity analyses and meta-regression to explore whether some characteristics could explain the results and evaluate robustness. Therefore, the results should be more reliable.

There were, however, several limitations of this meta-analysis. Firstly, although in the multivariable analysis we considered a multitude of lifestyle (including exercise) and dietary factors (such as smoking, alcohol). The possibility of residual confounding or confounding by unmeasured factors, which cannot be ruled out in any observational study, must be acknowledged. Second, the noticeable limitation of our study was the potential for bias due to inevitable measurement error, especially for individual with different serum uric acid levels. We attempted to reduce measurement error in adjusting for uric acid levels and using of generally serum urate concentration (6.8 mg/dl). Third, because we had no other sources of information other than medical records and/or death certificates for the identification of CHD events, we might have underestimated the morbidity and/or mortality of CHD. In addition, subclinical diseases at baseline might have distorted our risk estimate to some extent. Finally, the possible limitation is due to language bias. We attempted to minimize this bias by searching major electronic databases with no language restriction.

## Conclusions

This meta-analysis provides further evidence that hyperuricemia may increase the risk of CHD events, particularly CHD mortality in females. Future studies, preferably randomized controlled studies of agents that lower or prevent hyperuricemia, should explore whether hyperuricemia is a potentially modifiable risk factor for CHD.

## Additional Information

**How to cite this article**: Li, M. *et al.* Hyperuricemia and the risk for coronary heart disease morbidity and mortality a systematic review and dose-response meta-analysis. *Sci. Rep.* **6**, 19520; doi: 10.1038/srep19520 (2016).

## Supplementary Material

Supplementary Information

## Figures and Tables

**Table 1:** Characteristics of Cohort Studies of Hyperuricemia and Coronary Heart Disease.

Reference, publication (yr)	Country/Population	Participants (% males)	Age range or mean (yr)	Follow-up (yr)	Hyperuricemia Assessment (mg/dl)	No. of total CHD cases	Outcome Ascertainment	Variables controlled	Study quality
CHD INCIDENCE									
Freedman *et al.*^31^ 1995	United States	5421 (46)	25–74	13.5	7 (C); per 1 mg/dl increase	403 (M) 286 (F)	Based on hospital records and death certificates	Age, race, cholesterol, DBP, smoking, alcohol, education level, and use of antihypertensive and diuretic meds	9
Goldberg *et al.*^32^ 1995	United States	2710 (100)	55–64	23	6.8 (M)	352 (M)	Based on autopy reports and/or medical records such as EKGs and cardiac enzymes	Age	8
Liese *et al.*^33^ 1999	Germany/European	960 (100)	45–64	8	6.3 (M)	55 (M)	Based on medical records such as clinical symptoms, EKGs, cardiac enzymes, and autopsy reports	Age, alcohol, cholesterol/HDL ratio, HTN, smoking, BMI, education, and use of diuretics	9
Culleton *et al.*^34^ 1999	United States	6763 (45.5)	47 ± 15	17.4	6.8 (M) 6.3 (F)	394 (M) 223 (F)	Based on medical records such as clinical symptoms, EKGs, and cardiac enzymes	Age, BMI, SBP, use of antihypertensive and diuretic meds, DM, cholesterol, alcohol, smoking, LVH, and menopausal status	9
Moriarity *et al.*^35^ 2000	United States	13504 (43.7)	45–64	8	7.6 (M); 6.3 (F)	264 (M) 128 (F)	Based on medical records such as clinical symptoms, EKGs, and cardiac enzymes, and data on death certificates	Age, race, ARIC center, smoking, LDL, SBP, BMI, HDL, DM, waist/hip ratio, protein, TG, alcohol, and antihypertensive meds	9
Puddu *et al.*^36^ 2001	Italy/European	2469 (45.2)	35–74	6	7.3 (C)	68 (M) 41 (F)	Based on paper/phone questionnaires, EKGs, and medical records	Age, sex, SBP, cholesterol, DM, smoking, and BMI	9
Chien *et al.*^37^ 2005	China/Asian	3602 (47)	≥35	8.5	7.7 (M); 6.6 (F)	86	Based on death certificates and hospital records	Age, SBP, BMI, DM, cholesterol, smoking, and alcohol	9
Wheeler *et al.*^10^ 2005	Iceland/European	6042 (70.3)	56 ± 9	17.5	5.7 (M); 4.7 (F)	2080	Based on questionnaires, EKGs, and medical records	Age, smoking, SBP, cholesterol, BMI, TG, FEV1, and DM	9
Bos *et al.*^38^ 2006	Netherlands/European	4385 (35.4)	≥55	8.4	6.4 (M); 5.4 (F); 6.5 (C)	515	Based on ICD-9 codes on medical records	Age, sex, SBP, cholesterol, HDL, DM, smoking, diuretic use, and waist/hip ratio	9
Krishnan *et al.*^39^ 2006	United States	12866 (100)	46 ± 6	6.5	7.0 (M)	1108 (M)	Based on review of medical records such as EKGs and CABG surgery	Age, BP, cholesterol, serum creatinine, DM, smoking, BMI, family history of AMI, alcohol, aspirin and diuretic use	9
Baba *et al.*^40^ 2007	Japan/Asian	2024 (38.3	62 ± 9.9 (M); 63.2 ± 8.4 (W)	8	7.0 (C)	49	Based on self-reports, EKGs, and medical records	Age, sex, smoking, alcohol, DM, and fatty liver	9
Meisinger *et al.*^41^ 2008	Germany/European	3424 (100)	45–74	11.7	6.6 (M)	297 (M)	Based on the population based data from coronary event registry and death certificates	Age, smoking, alcohol, physical activity, HTN, BMI, DM, CRP dyslipidemia, creatinine, and diuretic use	9
Kavousi *et al.*^13^ 2012	Netherlands/European	5933 (40.6)	69.1 ± 8.5	6.8	5.0 (C)	347	Based on ICD-9 codes on medical records	Age, sex, BMI, SBP, treatment of HTN, total and HDL cholesterol levels, use of lipid-lowering medication, smoking, and DM	9
Storhaug *et al.*^14^ 2013	Norway/European	5700 (47.3)	55–75	12.5	6.0 (M); 5.7(F); per 1.5 mg/dl increase	659	Based on death certificates and hospital records	Age, BMI, SBP/DBP, HDL/TC, use of diuretics and other antihypertensive meds, smoking, physical activity, and renal factors	9
CHD MORTALITY									
Levine *et al.*^42^ 1989	United States	4825 (0)	45–64	11.5	Per 1 mg/dl increase	23 (F)	Based on ICD-8 codes on death certificates; autopsy and hospital reports if available	Age, weight, smoking, DBP, cholesterol, and antihypertensive meds	9
Fang *et al.*^43^ 2000	United States	5926 (45.6)	25–74	16.4	7.0 (M); 5.6 (F); Per 1 mg/dl increase	222 (M); 172 (F)	Based on ICD-9 codes on death certificates; hospital records if available	Age, cholesterol, race, BMI, smoking, alcohol, HTN, DM, and sex	9
Tomita *et al.*^44^ 2000	Japan/Asian	49413 (100)	25–60	5.4	6.5 (M)	85 (M)	Based on ICD-9 codes on health and pension records	Age	8
Eboule *et al.*^45^ 2001	Belgium/European	9701 (53.9)	25–74	10	7.0 (M); 5.4 (F)	150 (M) 51 (F)	Based on ICD-9 codes on hospital records	Age, DBP, education level, smoking, and alcohol (M); age, cholesterol, SBP, smoking, BMI, alcohol and DM (F)	9
Jee *et al.*^46^ 2004	Korea/Asian	22698 (100)	30–77	9	7.0 (M)	99 (M)	Based on ICD-9 and 10 codes from hospitalization records and death certificates	Age, HTN, DM, cholesterol, and smoking	9
Hakoda *et al.*^47^ 2005	Japan/ Asian	10615 (36.4)	49	24.9	7.0 (M); 6.0 (F)	177 (M) 250 (F)	Based on ICD-7 through 10 codes on death certificates	Age, BMI, smoking, alcohol, SBP, cholesterol, HTN, DM, kidney disease, malignant tumor, and estimated radiation dose from the atomic bombs	9
Baibas *et al.*^48^ 2005	Greece/European	1198 (42)	≥25	14	per 1 mg/1dl increase	34 (M) 33 (F)	Based on ICD-9 codes on death certificates	Age, body weight, smoking, alcohol, DM, SBP, cholesterol, village, TG, and educational level	9
Gerber *et al.*^49^ 2006	Israel/Asian	9125 (100)	49	23	5.6 (M)	830 (M)	Based on ICD-9 codes on death certificates and hospital records	Age, BMI, SBP, DM, cholesterol, smoking, and LVH on EKG	9
Krishnan *et al.*^50^ 2008	United States	9105 (100)	41–63	17	7.0 (M)	833 (M)	Based on ICD-9 and 10 codes on death certificates	Age, SBP/DBP, cholesterol, BMI, TG, serum creatinine, DM, alcohol, smoking, family history of AMI, aspirin and diuretic use	9
Strasak *et al.*^51^ (M) 2008	Austria/European	83683 (100)	41.6	12.4	6.8 (M)	844 (M)	Based on ICD-9 and 10 codes on death certificates autopsy records; if available	Age, BMI, cholesterol, SBP/DBP, TG, GGT, smoking, and year of examinations	9
Strasak *et al.*^52^ (F) 2008	Austria/European	28613 (0)	62.3	21	5.4 (F)	518 (F)	Based on ICD-9 and 10 codes on death certificates; autopsy records; if available	Age, BMI, cholesterol, SBP/DBP, TG, GGT, smoking, DM, occupational status, and year of examinations	9
Holme *et al.*^53^ 2009	Sweden/European	417734 (53)	30–85	11.8	6.1 (M); 5.5 (F); per 1 mg/dl increase	12286 (M) 4888 (F)	based on ICD-7, ICD-8, ICD-9, ICD-10, hospital records and the cause-of-death register	Age, sex, TC, TG, HTN, DM	9
Chen *et al.*^54^ 2009	China/Asian	90393 (46.3)	51.5	8.2	7.0 (M, F); per 1 mg/dl increase	286	Based on ICD-9 codes on death certificates	Age, sex, BMI, cholesterol, DM, TG, HTN, smoking, and alcohol	9
Chuang *et al.*^15^ 2012	China/Asian	128569 (46.6)	≥20	7.33	7.0 (M); 6.0 (F)	2049	Based on ICD-9 codes on death certificates and hospital records	Age, SBP/DBP, drugs using for HTN, diuretics using, BMI, TG, TC, DM, smoking, alcohol, physical activity, and working type	9
Zalawadiya *et al.*^16^ 2015	United States	11009 (unclear)	25–74	14.5	6.3 (C); per 1 mg/dl increase	458	Based on ICD-10 codes on death certificates; hospital records if available	Age, sex, race, BMI, SBP, smoking, HDL, cholesterol, antihypertensive meds, CRP, eGFR	9

CHD, coronary heart disease; M. male; F, female; C, combined; CT, computer tomography; LDL, low-density lipoprotein; TC, total cholesterol; TG, triglycerides HDL, high-density lipoprotein; BMI, body mass index; HTN, hypertension; DM, diabetes mellitus; EKG, electrocardiogram; CRP, c-reactive protein; GGT, gamma-glutamyl transferase; LVH, left ventricular hypertrophy; AMI, acute myocardial infarction; SBP, systolic blood pressure; DBP, diastolic blood pressure; BP, blood pressure; eGFR, estimated glomerular filtration rate; FEV1, forced expiratory volume in one second; CABG, coronary artery bypass graft; ICD, International Classification of Diseases; ARIC, Atherosclerosis Risk in Communities.
