# Supplementary material for: Hyperuricemia and the risk for coronary heart disease morbidity and mortality a systematic review and dose-response meta-analysis
Source: Sci Rep. 2016 Jan 27;6:19520. doi: 10.1038/srep19520 (PMC4728388; doi:10.1038/srep19520)
Supplement: Supplementary Information [file srep19520-s1.pdf]

MOOSE Checklist

Hyperuricemia and the risk for coronary heart disease morbidity and mortality

a systematic review and dose-response meta-analysis

Min Li[1†], Xiaolan Hu[2†], Yingli Fan[3], Kun Li[1], Xiaowei Zhang[1], Wenshang Hou[1] & Zhenyu Tang [1]

[1]Department of Neurology, The Second Affiliated Hospital to Nanchang University, Nanchang 330006, People's Republic of China;

[2]Department of Anaesthesiology, The Second Affiliated Hospital to Nanchang University, Nanchang 330006, People's Republic of China;

[3]Department of Cardiology, The Second Affiliated Hospital to Nanchang University, Nanchang 330006, People's Republic of China.

[†] Contributed equally

Correspondence and requests for materials should be addressed to Zhenyu Tang (zytang07016@sina.com).

Criteria		Brief description of how the criteria were handled in the meta-analysis
**Reporting of background should include**		
√	Problem definition	Cardiovascular disease is one of the most common noncommunicable diseases which is forecasted to be the major cause of morbidity and mortality in most developing nations by 2020. It has been estimated that 43.9% of the US population is projected to have some form of cardiovascular disease in 2030, this creates a public health crisis, especially in low and middle income countries. Approximately every 34 seconds, 1 American has a coronary event, and approximately every 1 minute 24 seconds, an American will die of one. The prevention of cardiovascular disease (especially coronary heart disease) is thus clearly a major public health issue. Among the novel risk factors for coronary heart disease, nutritional factors have aroused particular attention. Randomized controlled trials and prospective observational studies have been used to quantify the total effects of dietary habits on coronary heart disease. Although the effect of individual components or interactions between dietary habits is still largely unknown or even misconstrue actual total impact on vascular health, elevated serum uric acid levels may explain some of this harmful effect.
√	Hypothesis statement	Hyperuricemia is associated with increased risk of coronary heart disease morbidity and mortality.
√	Description of study outcomes	Coronary heart disease morbidity and mortality.
√	Type of exposure or intervention used	Hyperuricemia or elevated serum uric acid level.
√	Type of study designs used	We included (1) original studies (eg, not review articles, meeting abstracts, editorials, or commentaries); (2) prospective cohort design (eg, not cross sectional design, case-control design).
√	Study population	We placed no restriction.
**Reporting of search strategy should include**		
√	Qualifications of searchers	The credentials of the two investigators are indicated in the author list.
√	Search strategy, including time period included in the synthesis and keywords	PubMed from 1965 –August 23, 2015<br>EMBASE from 1974 –August 23, 2015<br>Keywords: ("hyperuricemia" OR "uric acid" OR "urate" and "coronary heart disease" OR "cardiovascular disease" OR "ischemic heart disease" OR "myocardial infarction" OR "coronary artery disease" OR "coronary disease" OR "angina pectoris" OR "unstable angina") AND ("follow-up studies" OR "prospective studies" OR "cohort studies" OR "longitudinal

		studies" OR "epidemiological studies" OR "observational studies").
√	Databases and registries searched	PubMed, EMBASE.
√	Search software used, name and version, including special features	We did not employ a search software. EndNote was used to merge retrieved citations and eliminate duplications
√	Use of hand searching	We hand-searched bibliographies of retrieved papers for additional references,
√	List of citations located and those excluded, including justifications	Details of the literature search process are outlined in the process of literature search and study selection. The citation list is available upon request
√	Method of addressing articles published in languages other than English	We placed no restrictions on language; local scientists fluent in the original language of the article were contacted for translation.
√	Method of handling abstracts and unpublished studies	We had contacted a few authors for unpublished studies on the association.
√	Description of any contact with authors	We contacted authors who had conducted multivariate analysis with uric acid levels as a covariate, but the outcome of interest was not coronary heart disease.
**Reporting of methods should include**		
√	Description of relevance or appropriateness of studies assembled for assessing the hypothesis to be tested	Detailed inclusion and exclusion criteria were described in the methods section.
√	Rationale for the selection and coding of data	Data extracted from each of the studies were relevant to the population characteristics, definition of hyperuricemia, and possible effect modifiers of the association.
√	Assessment of confounding	Restricted the analysis to not adjusted estimates. Conducted sensitivity analyses by eliminating studies that had only age-adjusted for possible confounders and others.
√	Assessment of study quality, including blinding of quality assessors; stratification or regression on possible predictors of study results	The Newcastle-Ottawa Scale (NOS) was used to assess the quality of studies. The quality of cohort studies were evaluated in the following three major components: selection of the study group (0-4 stars), quality of the adjustment for confounding (0-2 stars) and assessment of outcome in the cohorts (0-3 stars). A higher score represents better methodological quality. The full score was 9 stars. Studies were graded as the high-quality if they met ＞8 awarded stars.
√	Assessment of heterogeneity	Heterogeneity of the studies were explored within two types of study designs using Cochrane's Q test of heterogeneity and I^2 statistic that provides the relative amount of variance of the summary effect due to the between-study heterogeneity.
√	Description of statistical methods in sufficient detail to be replicated	Description of methods of meta-analyses, sensitivity analyses, subgroup analyses, meta regression and assessment of publication bias are detailed in the methods.

√	Provision of appropriate tables and graphics	We included 1 flow chart, several summary tables and figures.
**Reporting of results should include**		
√	Graph summarizing individual study estimates and overall estimate	Figure 2, 3 and 4, Supplemental figures F and L
√	Table giving descriptive information for each study included	Table 1 and Supplemental tables A, B and C
√	Results of sensitivity testing	Supplemental table C, Supplemental figures B and I
√	Indication of statistical uncertainty of findings	95% confidence intervals were presented with all summary estimates, I^2 values and results of sensitivity analyses
**Reporting of discussion should include**		
√	Quantitative assessment of bias	Subgroup analyses indicate heterogeneity in strengths of the association due to most common biases in cohort studies.
√	Justification for exclusion	We excluded animal studies, clinical trials, commentaries and letters without sufficient data.
√	Assessment of quality of included studies	We discussed the results of the subgroup analyses, and potential reasons for the observed heterogeneity.
**Reporting of conclusions should include**		
√	Consideration of alternative explanations for observed results	We discussed that potential unmeasured confounders such as other chronic diseases may have caused residual confounding, but the measured factors that are correlated with such confounders would have mitigated the bias.<br>We noted that the variations in the strengths of association may be due to true population differences, or to differences in quality of studies.
√	Generalization of the conclusions	Our meta-analysis suggests that hyperuricemia is associated with a significantly increased risk of coronary heart disease. In addition, the dose-response relations also indicate that relatively high serum uric acid may still increase risk of coronary heart disease mortality among females.
√	Guidelines for future research	We recommend further evidence from preferably larger sample sizes and longer follow-up term studies should be warranted to explore what effect of urate-lowering therapy on coronary heart disease.
√	Disclosure of funding source	No separate funding was necessary for the undertaking of this systematic review.

Additional file
Appendix figures information
Supplemental fig A. Egger’s funnel plots for detection of publication bias for coronary heart disease morbidity.

Supplemental fig B. Filled Begg’s funnel plot for publication bias in studies for coronary heart disease morbidity.

Supplemental fig C. Dose-response analyses of SUA level and risk of coronary heart disease morbidity in males. SUA: serum uric acid

Supplemental fig D. Dose-response analyses of serum uric acid level and risk of coronary heart disease morbidity in females.

Supplemental fig E. Random effects analysis of multivariate risks of coronary heart disease morbidity associated with an increase of 1 mg/dl in serum uric acid level.

Supplemental fig F. Begg’s funnel plot for publication bias in studies for coronary heart disease mortality.

Supplemental fig G. Egger’s funnel plots for detection of publication bias for coronary heart disease mortality.

Supplemental fig H. Dose-response analyses of serum uric acid level and risk of coronary heart disease mortality in males.

Supplemental fig I. Dose-response analyses of serum uric acid level and risk of coronary heart disease mortality in females.

Supplemental fig J. Random effects analysis of multivariate risks of coronary heart disease mortality associated with an increase of 1 mg/dl in serum uric acid level.

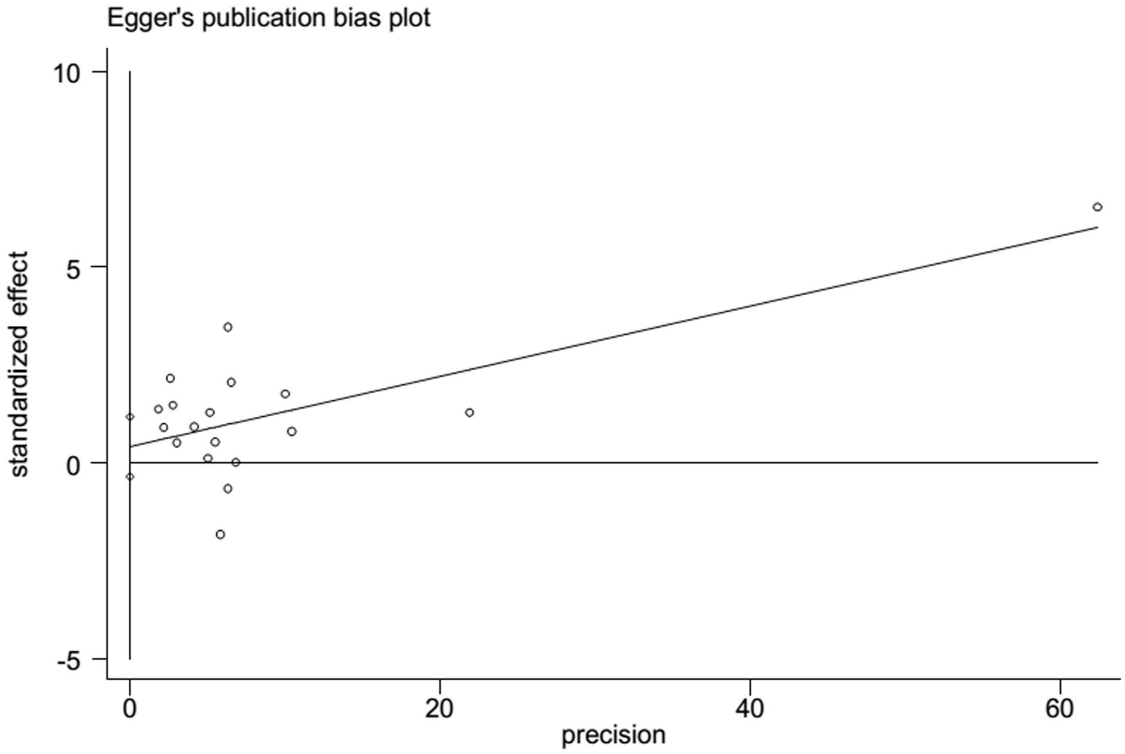

Supplemental fig A

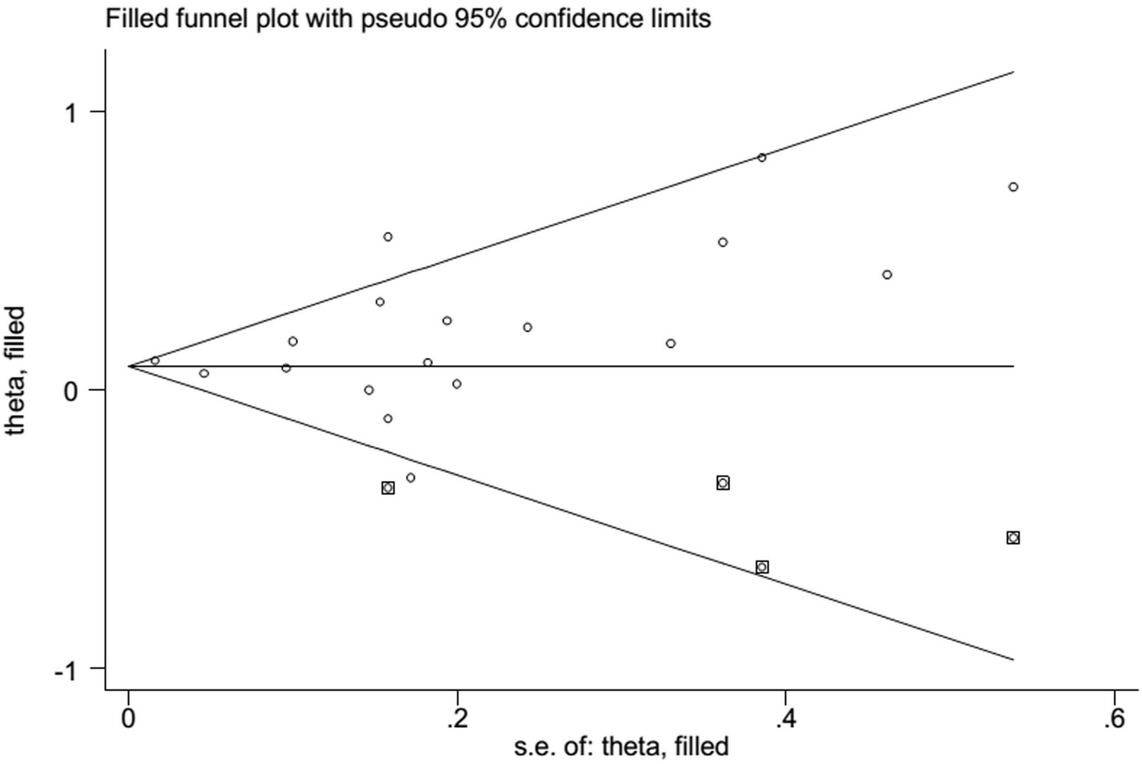

Supplemental fig B

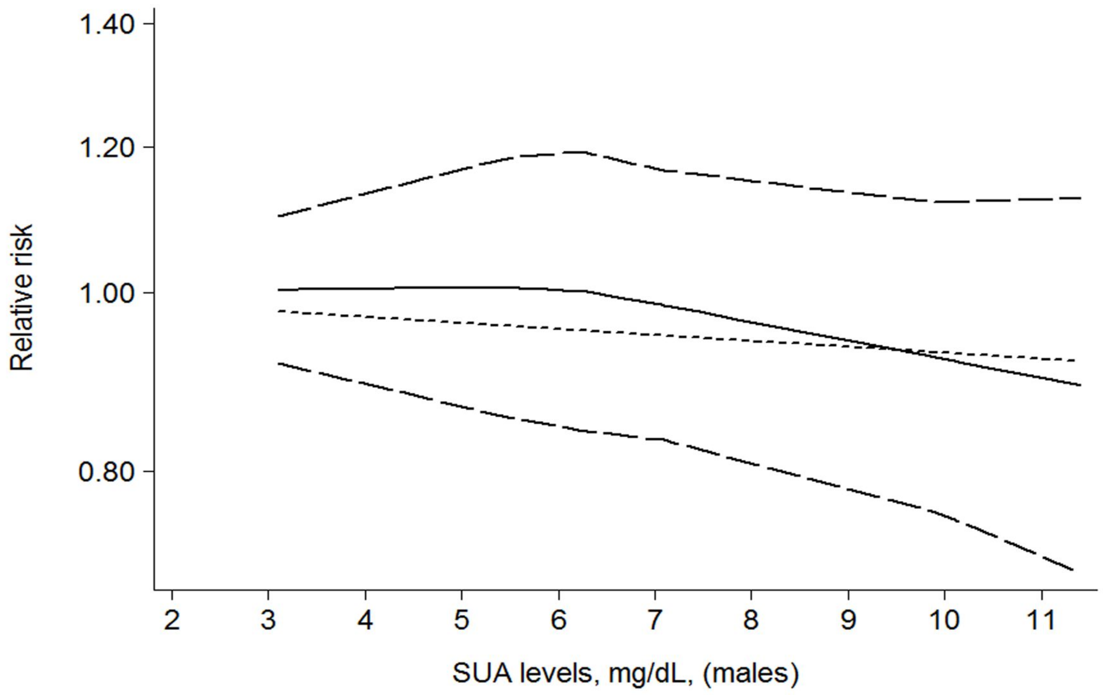

Supplemental fig C

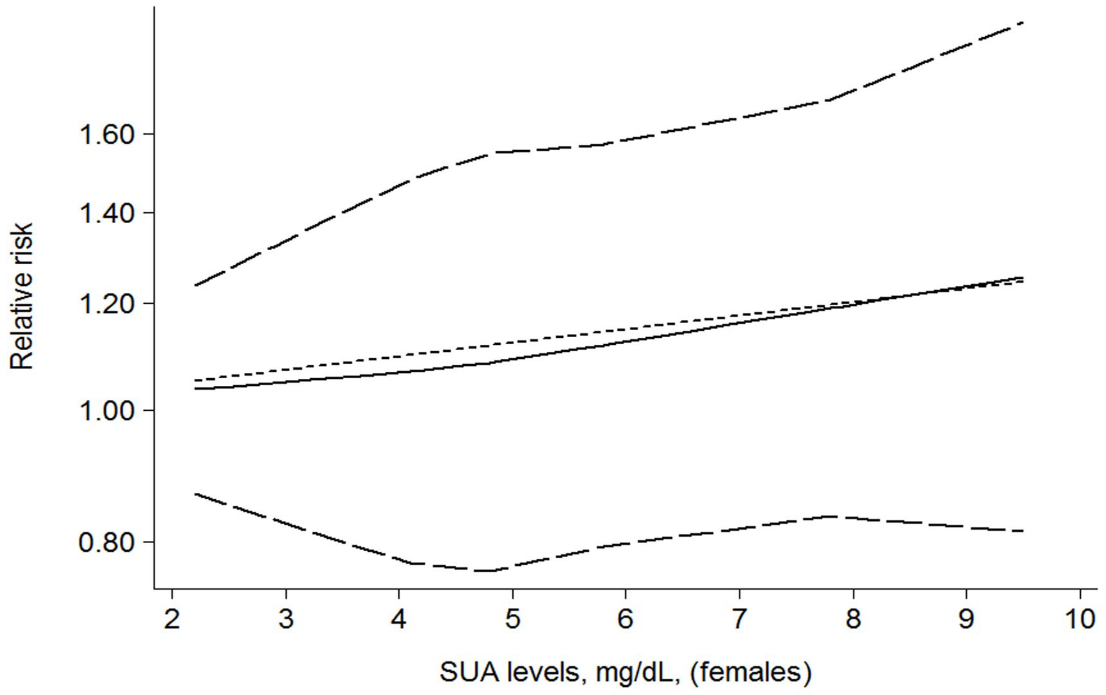

Supplemental fig D

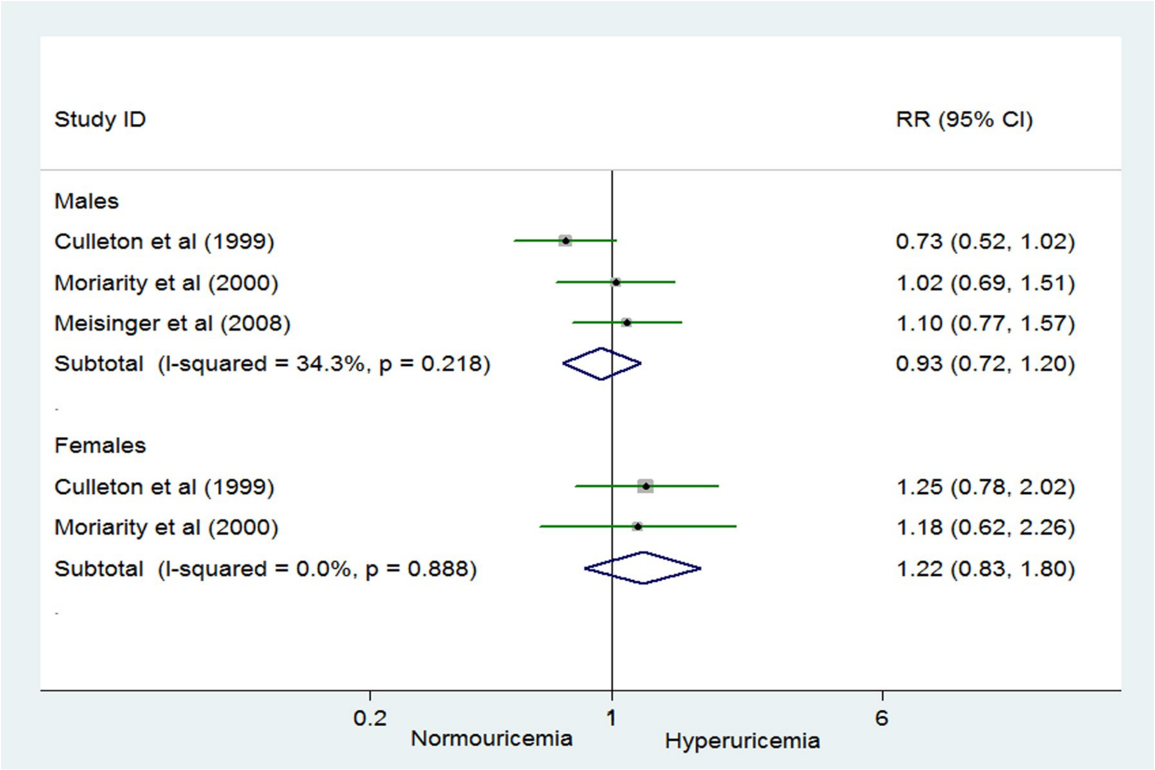

Supplemental fig E

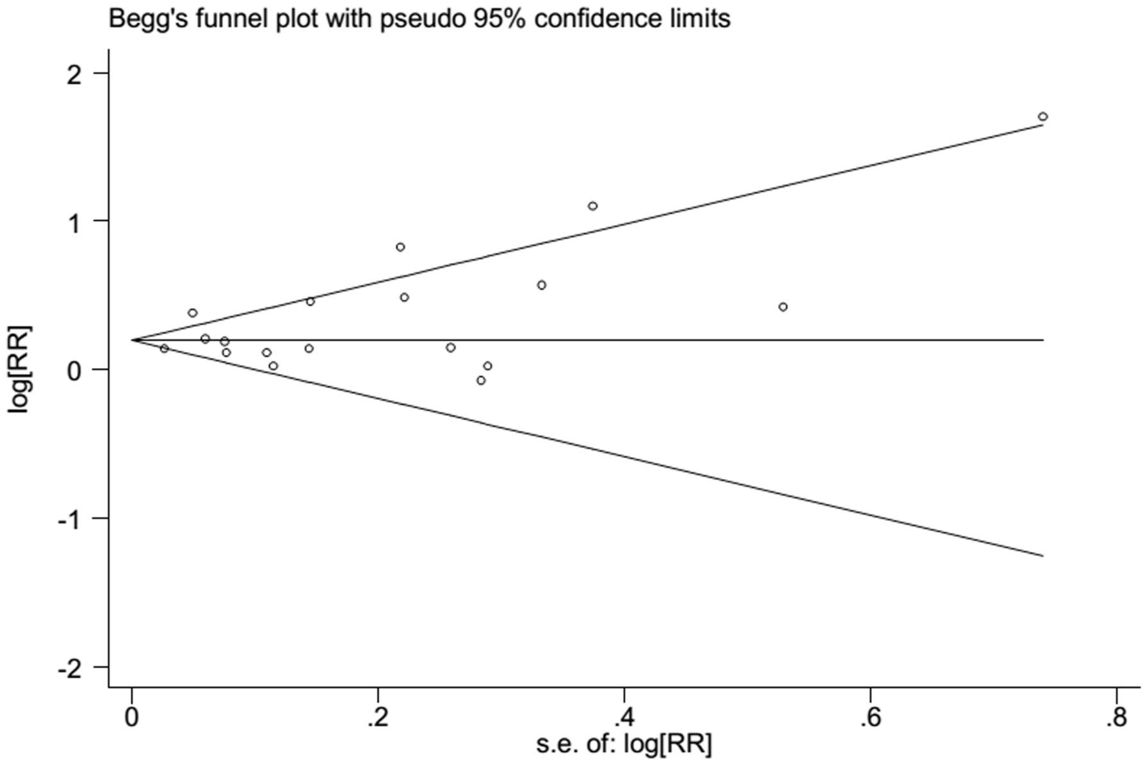

Supplemental fig F

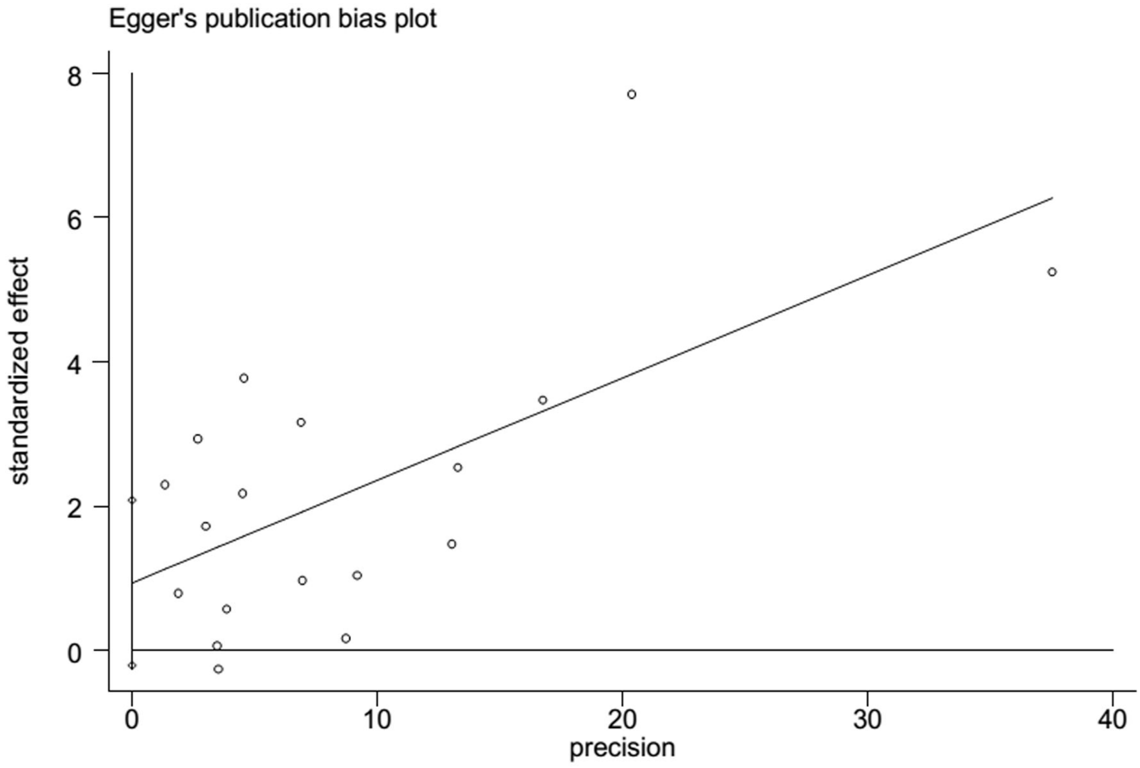

Supplemental fig G

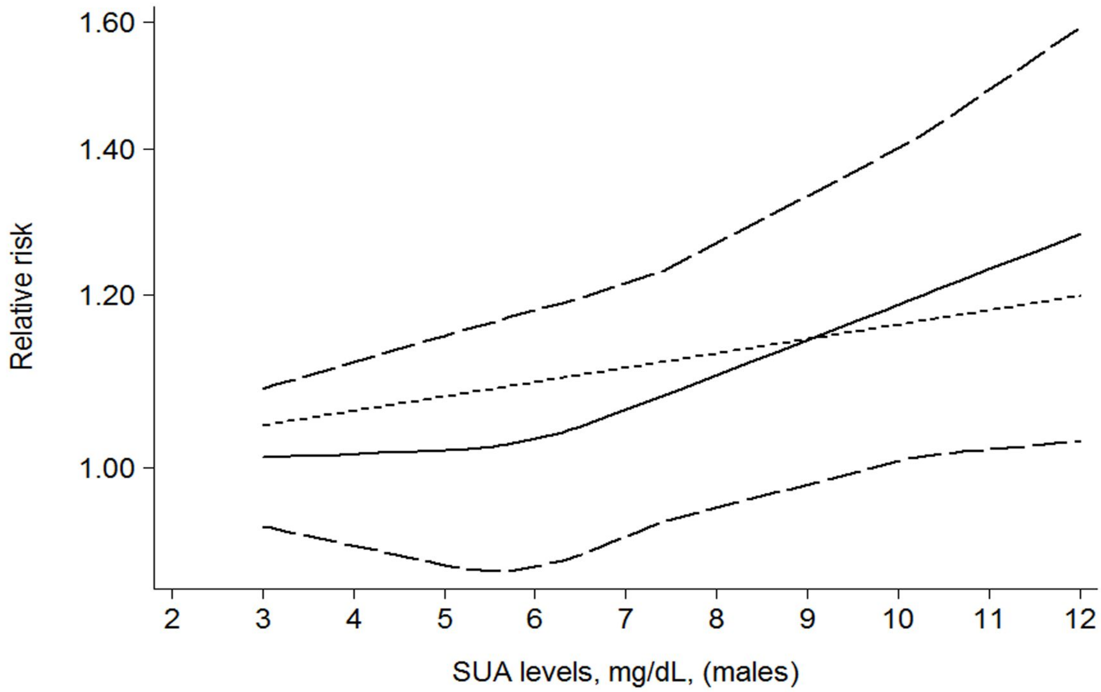

Supplemental fig H

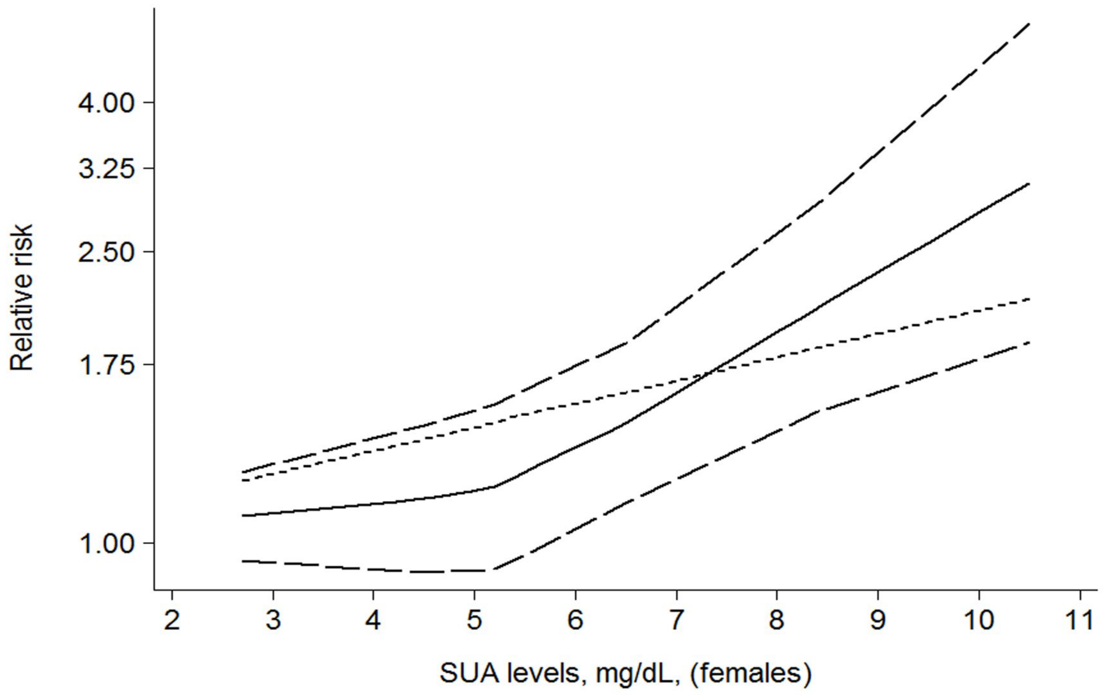

Supplemental fig I

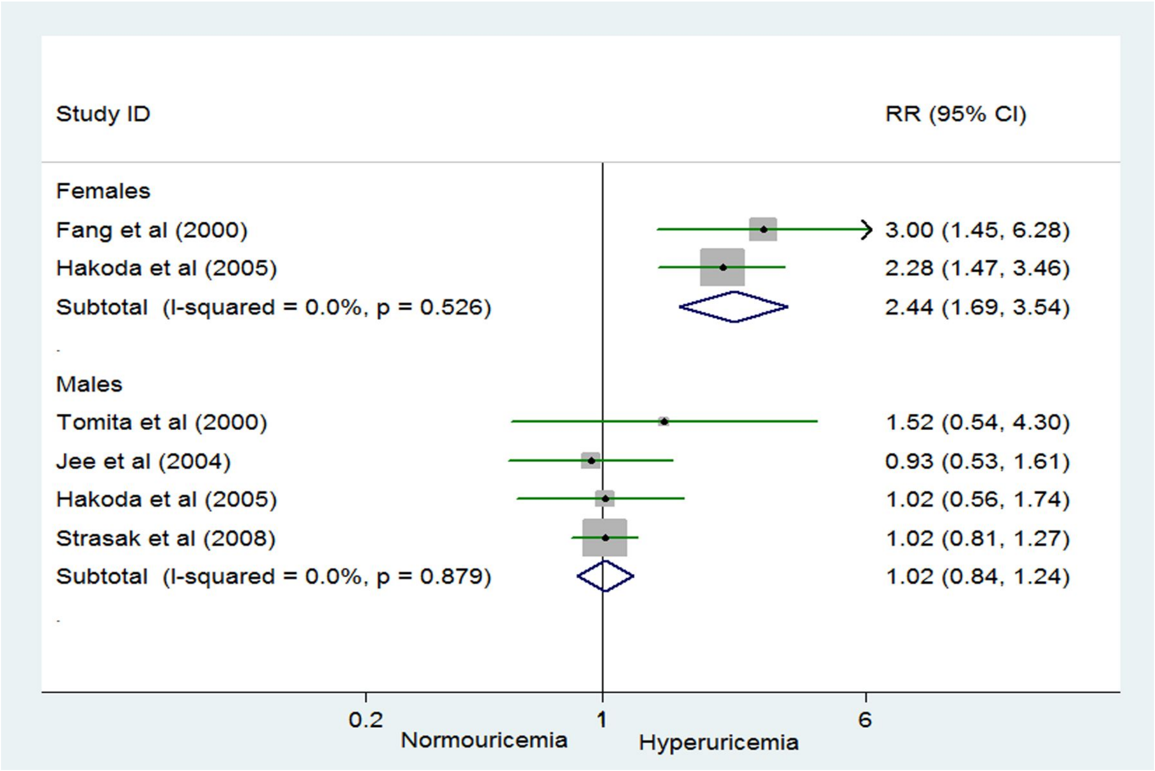

Supplemental fig J

Appendix tables information

Supplemental table A Quality assessment of included studies by Newcastle-Ottawa Scale.

Supplemental table B Effect of study variables by meta-regression.

Supplemental table C Subgroup analyses of hyperuricemia and risk of coronary heart disease morbidity and mortality.

Supplemental table A Quality assessment of included studies by Newcastle-Ottawa Scale.

	Selection				Comparability		Outcome			
Reference, publication (yr)	Representativeness of exposed cohort	Selection of non-exposed cohort	Exposure Ascertainment	Outcome present at start of study	Study controls for age	Study controls for any additional important factor	Assessment of Outcome	Length of follow-up	Adequacy of follow-up	Score
**CHD INCIDENCE**										
Freedman et al[31] 1995	★	★	★	★	★	★	★	★	★	9
Goldberg et al[32] 1995	★	★	★	★	★		★	★	★	8
Liese et al[33] 1999	★	★	★	★	★	★	★	★	★	9
Culleton et al[34] 1999	★	★	★	★	★	★	★	★	★	9
Moriarity et al[35] 2000	★	★	★	★	★	★	★	★	★	9
Puddu et al[36] 2001	★	★	★	★	★	★	★	★	★	9
Chien et al[37] 2005	★	★	★	★	★	★	★	★	★	9
Wheeler et al[10] 2005	★	★	★	★	★	★	★	★	★	9
Bos et al[38] 2006	★	★	★	★	★	★	★	★	★	9

Krishnan et al[39] 2006	★	★	★	★	★	★	★	★	★	9
Baba et al[40] 2007	★	★	★	★	★	★	★	★	★	9
Meisinger et al[41] 2008	★	★	★	★	★	★	★	★	★	9
Kavousi et al[13] 2012	★	★	★	★	★	★	★	★	★	9
Storhaug et al[14] 2013	★	★	★	★	★	★	★	★	★	9
**CHD MORTALITY**										
Levine et al[42] 1989	★	★	★	★	★	★	★	★	★	9
Fang J et al[43] 2000	★	★	★	★	★	★	★	★	★	9
Tomita et al[44] 2000	★	★	★	★	★		★	★	★	8
Eboule et al[45] 2001	★	★	★	★	★	★	★	★	★	9
Jee et al[46] 2004	★	★	★	★	★	★	★	★	★	9
Hakoda et al[47] 2005	★	★	★	★	★	★	★	★	★	9
Baibas et al[48] 2005	★	★	★	★	★	★	★	★	★	9

Gerber et al[49] 2006	★	★	★	★	★	★	★	★	★	9
Krishnan et al[50] 2008	★	★	★	★	★	★	★	★	★	9
Strasak et al[51] (M) 2008	★	★	★	★	★	★	★	★	★	9
Strasak et al[52] (F) 2008	★	★	★	★	★	★	★	★	★	9
Holme et al[53] 2009	★	★	★	★	★	★	★	★	★	9
Chen et al[54] 2009	★	★	★	★	★	★	★	★	★	9
Chuang et al[15] 2012	★	★	★	★	★	★	★	★	★	9
Zalawadiya et al[16] 2014	★	★	★	★	★	★	★	★	★	9

CHD, coronary heart disease; M. male; F, female.

Supplemental table B Effect of study variables by meta-regression.

	CHD	Morbidity	CHD	Mortality
	Coefficient	95% CI	Coefficient	95% CI
Sex	0.03	-1.29-1.35	0	-1.54-1.54
Duration of follow-up	-0.22	-2.06-1.61	0.21	-1.75-2.18
Geographical area	-0.26	-1.96-1.44	0.35	-1.73-2.43
Study quality	-0.09	-3.05-2.88	-0.07	-3.55-3.42

CHD, coronary heart disease; CI, confidence interval.

Supplemental table C Subgroup analyses of hyperuricemia and risk of CHD morbidity and mortality.

Subgroup	CHD Morbidity				CHD Mortality			
	RR (95% CI)	I^2 %	P_{het}	P_{eff}	RR (95% CI)	I^2 %	P_{het}	P_{eff}
Duration of follow-up (years)								
＞10	1.05 (0.97-1.14)	5.2	0.387	0.212	1.30 (1.14-1.48)	75.8	0.000	0.000
≤10	1.23 (1.08-1.41)	46.5	0.044	0.002	1.23 (1.11-1.37)	14.5	0.319	0.000
Study quality								
Score = 9	1.12 (1.04-1.21)	40.2	0.044	0.003	1.27 (1.16-1.39)	66.9	0.000	0.000
Score ＜9	1.28 (0.88-1.87)	-	-	0.202	1.52 (0.54-4.29)	-	-	0.429
Geographical area								
United States	1.07 (0.93-1.23)	27.7	0.227	0.331	1.46 (0.99-2.14)	63.3	0.043	0.058
Asian	1.97 (1.18-3.27)	0	0.778	0.009	1.22 (1.09-1.37)	33.2	0.163	0.001
European	1.15 (1.03-1.28)	46.7	0.059	0.015	1.33 (1.12-1.59)	83.2	0.000	0.001
Number of participants								
＞5000	1.08 (1.02-1.13)	12.9	0.327	0.004	-	-	-	-
≤5000	1.35 (1.18-1.53)	4.7	0.396	0.000	-	-	-	-
Sex								
Males	1.09 (1.02-1.17)	20.9	0.251	0.009	1.15 (1.11-1.20)	0	0.601	0.000
Females	1.22 (0.99-1.51)	49.2	0.08	0.058	1.59 (1.31-1.91)	65.5	0.008	0.000
Combined	1.18 (0.84-1.66)	65.2	0.057	0.342	1.16 (0.70-1.93)	-	-	0.566

CHD, coronary heart disease; RR, relative risk; CI, confidential interval; P_{eff}, P value of pooled effect; P_{het}, P value of heterogeneity test.
